# Process-Driven Inference of Biological Network Structure: Feasibility, Minimality, and Multiplicity

**DOI:** 10.1371/journal.pone.0040330

**Published:** 2012-07-18

**Authors:** Guanyu Wang, Yongwu Rong, Hao Chen, Carl Pearson, Chenghang Du, Rahul Simha, Chen Zeng

**Affiliations:** 1 Department of Physics, George Washington University, Washington, D.C., United States of America; 2 Department of Mathematics, George Washington University, Washington, D.C., United States of America; 3 Department of Computer Science, George Washington University, Washington, D.C., United States of America; 4 Department of Physics, Huazhong University of Science and Technology, Wuhan, China; Queen’s University Belfast, United Kingdom

## Abstract

A common problem in molecular biology is to use experimental data, such as microarray data, to infer knowledge about the structure of interactions between important molecules in subsystems of the cell. By approximating the state of each molecule as “on” or “off”, it becomes possible to simplify the problem, and exploit the tools of Boolean analysis for such inference. Amongst Boolean techniques, the *process-driven* approach has shown promise in being able to identify putative network structures, as well as stability and modularity properties. This paper examines the process-driven approach more formally, and makes four contributions about the computational complexity of the inference problem, under the “dominant inhibition” assumption of molecular interactions. The first is a proof that the feasibility problem (does there exist a network that explains the data?) can be solved in polynomial-time. Second, the minimality problem (what is the smallest network that explains the data?) is shown to be NP-hard, and therefore unlikely to result in a polynomial-time algorithm. Third, a simple polynomial-time heuristic is shown to produce near-minimal solutions, as demonstrated by simulation. Fourth, the theoretical framework explains how multiplicity (the number of network solutions to realize a given biological process), which can take exponential-time to compute, can instead be accurately estimated by a fast, polynomial-time heuristic.

## Introduction

A central theme in molecular biology is to understand the complex network of interactions between biomolecules and how those interactions contribute to higher-level biological function [1]–[6]. This problem belongs to the broader field of network analysis: graph theoretic representation of objects in physics, biology, sociology, etc; and the categorization and analysis of graph properties [7], [8]. Many important network classes have been well studied, such as random networks [9], small-world networks [10], and scale-free networks [11]. Many important measures and concepts have been defined to characterize network structures, such as degree distribution [12], clustering coefficient [7], the Estrada index [8], entropy-based molecular descriptors [13]–[16], and network motif [17]. These structural approaches have been used in studying biological networks. For example, topological properties such as scale-free, power-law degree distribution [18] have been found for biomolecular networks. The implications of scale-freeness on the robustness and evolvability of genetic regulatory networks have been studied in [19].

Against the backdrop of the success of structural network analysis is the disappointing reality that for many subsystems in the cell, very little is known about the network structure. Instead, what is observable from data is whether or not the molecules are active at a given moment when a “snapshot” (such as a microarray sample) is taken. Given a collection of such snapshots that represent the dynamics of the system, one major goal is to address the *network inference problem*: what possible network structures could have resulted in the given dynamics? The network inference problem is made challenging because of the variety of molecule-to-molecule interactions and by the sheer number of molecules in even small subsystems of the larger cell. As reviewed in [20], many important inference approaches have recently emerged, which include clustering-based (e.g., [21]), Boolean-based (e.g., [22]), Bayesian-based (e.g., [23]), *in silico*-based (e.g., [20]), and information-theory-based methods. For example, the algorithm C3NET, based on the estimation of mutual information, is a successful example of information-theory-based methods [24]. Boolean-based analysis is relatively simple and might be suitable to handle large-scale data [25]–[27]. In [28], the authors investigated the effects of discretisation methods, biological constraints, and stringency of Boolean function assignment on the performance of Boolean network, by using performance indices such as accuracy, precision, specificity and sensitivity. These performance indices indicate the correctness of the inferred network based upon the matches between the inference and reference networks. For example, sensitivity was defined as 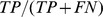, where 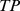 (true positive) is the number of network connections that were correctly predicted and 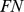 (false negative) is the number of network connections that were deemed as disconnected. They found that biological constraints have pivotal influence on the network performance over the other factors.

In [29], the Boolean network inference problem has been made computationally tractable by some simplifying assumptions. The goal of this paper is to examine this computational complexity more formally. In particular, we show that the existence problem (is there a network that explains the dynamics?) can be solved quickly, in polynomial-time whereas the minimality problem (what is the smallest network that explains the dynamics?) is NP-hard. Fortunately, a simple heuristic, which while not guaranteed to find the minimal network, does appear to find near-minimal networks very efficiently. Furthermore, our formulation also sheds light on the exponential counting problem of *multiplicity* (also called *designability* by some authors), the number of networks that explain a given dynamics.

There are two simplifying assumptions we make in this paper. One of these is the Boolean molecule-state assumption adopted by most prior work in the literature on Boolean-network models [1], [30]–[32]: at any given moment, a given biomolecule is either “on” (active or highly-expressed) or “off” (inactive or inhibited), and molecules stimulate or inhibit other molecules. The successive application of stimulatory or inhibitory interaction results in the next state for each molecule, and in this manner, the system evolves from state to state. The second assumption we make is the *dominant inhibition* assumption [30], [33], [34], in which any competition between inhibition and stimulation results in inhibition. These two assumptions allow an analysis that is entirely Boolean in nature and let us, after some algebraic reduction, exploit fast algorithms for the class of Boolean expressions called Horn formulas. The satisfiability of a Horn formula is known to be determinable in polynomial-time [35], [36].

The complexity results in this paper imply that large systems can be solved efficiently, in polynomial-time, when the goal is to obtain solution networks or to obtain a characterization of the class of network solutions. On the other hand, when the goal is to find the smallest network, the problem remains hard; however, a simple heuristic approach appears to work well in practice although no theoretical guarantees can be made about the minimality of the heuristic solutions. In either case, both the existence problem and the minimization problem reveal useful characteristics of the network. These include the network “backbone,” a list of edges that must be present in all network solutions, as well as additional groups of edges called network modules or network motifs that are thought to be an important organizing principle for biological networks [37].

The results of the paper are organized as follows. In Section 1, we introduce the Boolean network model and process-driven analysis. In Section 2, we study the feasibility problem and its applications. In Section 3, we discuss the computational complexity of finding a minimal network and develop an algorithm to find an approximated minimal network. In Section 4, we develop a polynomial-time algorithm to estimate multiplicity. Technical details and illustrative examples are further provided in File S1.

## Results

### 1. Boolean Network Model

The starting point for our model is a collection of 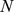 interacting molecules, each of which at any given time is modeled as either “on” or “off.” Let 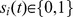 denote the state of molecule 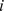 and 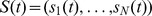 the state of the system at time 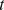. Here, the change of state is assumed to occur over a large enough time interval for biochemical reactions to complete, and thus time is assumed to be discrete: 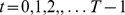. A sequence of such system states, 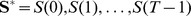 is what we term a *Boolean process*. Intuitively, in biological terms, a Boolean process corresponds to discretized time-course data. Thus, a sequence of microarray snapshots taken for a system of molecules over a time course can be converted into this Boolean form by noting which molecules are active and which are not.

In [3], Li *et al.* introduced a specific type of Boolean network model to determine the next state of a particular node 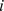 from the current state:
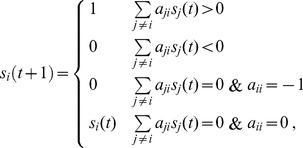
(1)where 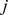 ranges over 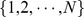. Each non-diagonal entry, 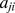 (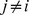), takes the value 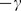, 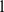, or 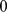, depending on whether node 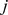 inhibits, stimulates, or does not interact with, node 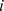. The diagonal entries, 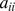, take the value 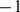 (degradation) or 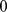 (no degradation). The inclusion of degradation allows the model to determine whether degradation plays an important role in the dynamics. Note: we have not explicitly included the case that node 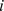 is self-activating (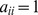), because a self-activating node can be replaced by two different nodes with one activating the other.

The parameter 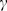 models the relative dominance of inhibition over stimulation. Since we assume inhibition dominates stimulation for most biomolecular interactions, 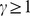 in our model. Simulation results reported in [29] show that for the budding yeast network, the cases 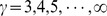 produce exactly the same dynamics and are only slightly different from the cases 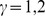; for the fission yeast network, the cases 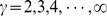 produce exactly the same dynamics and are only slightly different from the case 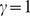. We therefore follow the “dominant inhibition” assumption [30], [33], [34] by setting 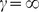. This assumption renders a simpler, purely logical representation of Eq. (1), namely:
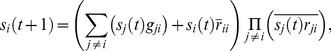
(2)where addition represents the Boolean operator OR, multiplication represents AND, and the bar on a variable represents NOT. The Boolean variable 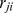 represents a putative inhibitory edge (conventionally drawn in red color) from node 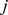 to node 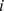. The Boolean variable 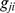 represents a putative stimulatory edge (conventionally drawn in green color) from node 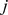 to node 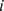. Note that each 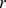 or 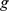 is modulated by an 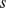-variable because edge 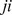 is active only when 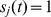. For Eq. (2), one additional constraint, 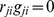, is imposed to exclude solutions where an edge interaction is both inhibitory and stimulatory, a possibility absent in Eq. (1). However, under “dominant inhibition” condition, such a solution with an edge being both inhibitory and stimulatory produces exactly the same dynamics as if the edge were only inhibitory because the inhibition dominates over the stimulation. Thus this constraint will only come into play in Section 4 on solution multiplicity, and the Boolean variables 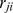 and 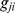 will be treated as independent for the rest of the paper.

To understand Eq. (2), consider just one of the four possible transitions for node 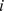 at some time step 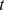, for example, 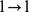. This transition requires either at least one active stimulatory edge, or no active self degradation if no active stimulatory edge is present, and no active inhibitory edge overall. These three conditions correspond precisely to the three terms 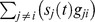, 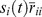, and 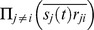 in Eq. (2) respectively. To prove Eq. (2), one similarly completes the analysis for the remaining three possible transitions of 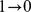, 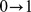, and 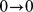.

### 2. Feasibility: Does a Solution Exist?

#### 2.1. Simplification

Equation (2), while compact, is somewhat inconvenient for determining the feasibility of a Boolean process. By a simplification procedure (see File S1 A), Eq. (2) is instead converted into four equations each corresponding to a type of state transition of node 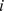:
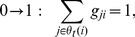
(3)

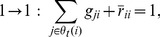
(4)

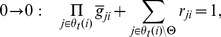
(5)

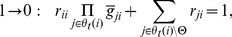
(6)where 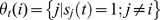 represents the set of nodes other than 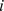 that are active at time 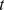, 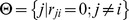 represents the set of nodes that are found not inhibiting node 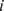 (see File S1 A as to how these nodes are identified), and 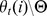 represents the set of nodes in 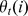 but with the ones in 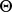 excluded.

#### 2.2. Conjunctive normal form

Equations (3)–(6) represent a Boolean satisfiability (SAT) problem – the problem of deciding whether a setting of variables 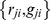 can satisfy a Boolean statement. The general satisfiability problem is usually given in the so-called Conjunctive Normal Form (CNF) [38]. A CNF formula or statement consists of a number of clauses in conjunction, and where each clause features variables or their complements with the OR operator. While a solution to the satisfiability problem of a general CNF statement is NP-complete [39], there are some types of CNF that lend themselves to a polynomial solution: a CNF in Horn-clause form can be solved in polynomial time [35], [36].

First, note that the first two equations above are already in the form of CNF clauses. The other two are not because they feature products that are not allowed inside clauses. However, they can be multiplied out to create CNF clauses as follows. Treating the entire sum of 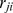
’s with the symbol 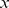, each equation is of the form.
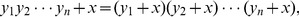
(7)where 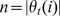. Then, with 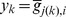 (for 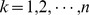) and 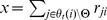, one has



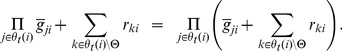
(8)Note that the conversion takes 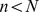 steps, which is clearly polynomial. In summary, we have the following new forms of equations:
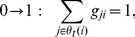
(9)

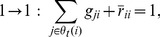
(10)

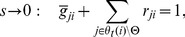
(11)

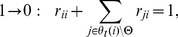
(12)where 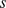 can be either 0 or 1.

#### 2.3. Horn formula

With the equations transformed into the conjunctive normal form, one can apply a SAT algorithm to determine whether the given Boolean process is feasible or not. Although a general SAT problem is NP-complete [40], some special class of SAT problem can be solved in polynomial time. For example, the 2-SAT problem (the number of literals in a clause is limited to 2), is a polynomial time problem [41]. Another important example, relevant to our case, is the HORNSAT problem.

In mathematical logic, a Horn clause is a clause with at most one positive literal. The positive literal is called the head and the negative literals form the body of the clause. The Horn clause form is named after the logician Alfred Horn, who first pointed out the significance of such clauses in [42]. Indeed, Horn clause forms have played a basic role in logic programming and are important for constructive logic [43]–[45]. A Horn formula is a propositional formula formed by the conjunction of Horn clauses. In formal logic, Horn-satisfiability, or HORNSAT, is the problem of deciding whether a given Horn formula is satisfiable.

Because HORNSAT can be determined in polynomial-time [35], [36], [45], this means that the network existence problem (for a given dynamics) can be solved in polynomial-time, as discussed below.

We now show how to convert the particular CNF formula for our Boolean network into a Horn formula. First, recall that we have formed a set of nodes 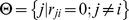. In the following, we let lowercase letters 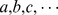 denote nodes belonging to 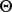; leave node 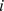 as it is; and let uppercase letters 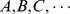 denote the other nodes. With these new notations, the left hand side of Eqs (9)–(12) can be represented by the following clause forms:
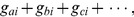
(13)

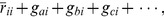
(14)

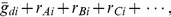
(15)

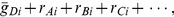
(16)

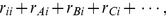
(17)where expressions (13), (14), and (17) are from Eqs. (9), (10), and (12), respectively; expressions (15) and (16) are from Eq. (11). Note that the used subscripts do not correspond to the actual nodes; they only indicate to which set (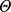 or not 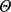) the nodes belong. For example, the subscript 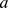 in Eq. (13) does not necessarily equal the subscript 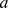 in Eq. (14), and similarly the subscript 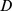 in Eq. (16) is allowed to equal the subscript 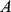 in the same equation.

For the nodes 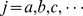, we define new variables.
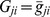
to replace 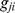. That is, we shall use the four variables 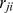, 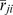, 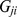, and 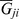 for 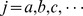. For the nodes 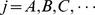, we define new variables



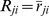
to replace 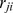. That is, we shall use the four variables 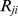, 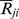, 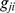, and 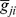 for 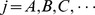. The clauses (13)–(17) now turn into



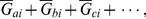
(18)

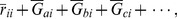
(19)

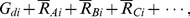
(20)

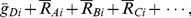
(21)

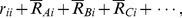
(22)which are in Horn-clause form.

To summarize, the simplification (to CNF) and conversion steps result in a Horn formula that is solvable in polynomial time.

#### Theorem

Given a Boolean process, the feasibility problem (the problem of determining whether or not the process can be realized by a network based on the model Eq. (2)), can be solved in polynomial time.

File S1 B and C provide two examples for CNF and Horn formula conversions. A different proof of the above theorem using Eq. (2) directly is also given in File S1 D.

### 3. Finding a Minimal Network

Minimality is an important concept in studying genetic networks [46], [47], biochemical networks [48], and cell signaling networks [49], [50], for example. In the context of this paper, it refers to making the smallest number of positive assignments (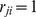 or 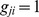) necessary for the Boolean equations to be satisfied. Because each such an assignment corresponds to an interaction edge in the biomolecular network, a minimal number of positive assignments corresponds to a minimal network –– a network that can realize the same biological function but has the smallest number of edges [51]. For example, the budding yeast cell cycle process in [29] can be produced by 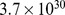 networks, according to the present model Eq. (2). Among these network solutions, there are 40,300 networks that only have 23 edges. The remaining networks all have more than 23 edges. The 40,300 networks are therefore minimal networks because they have the smallest number of edges. The minimality of the budding yeast cell cycle process is therefore 23.

It is important to identify these minimal networks. First, the smallest network that “explains” a biological process helps identify the core relationships between molecules that *must be present* for the biological process to function. Second, in a real biological network, the remaining edges beyond the minimal network often confer some functionality orthogonal to the biological process. For example, for the cell-cycle process, the non-minimal edges were found to confer stability properties [29]. Therefore, identifying minimal networks may well be the key to understanding the important elements of a biological process.

In general, a minimal network solution is difficult to find by chance, because the minimal solutions occupy only a tiny fraction of the whole solution space (e.g., 40,320 out of 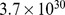 for the case of budding yeast cell cycle process). Perhaps for this reason, a large number of algorithms have been developed in the area of learning sparse Boolean functions [48], [52]–[55]. These algorithms usually take minimality as a constraint in learning.

From the computational complexity viewpoint of this paper, one would like to know whether it is even computationally feasible to compute a minimal solution in reasonable time. Some early studies [25], [27], [56] have considered and established the connection between the minimal Boolean network inference problem and the minimal set cover (MSC) problem. For the present Boolean model, we show that the minimal network problem is of equal complexity to the MSC problem, which is well-known to be NP-complete. The comparison also enables the development of a fast heuristic that, when evaluated with randomly-generated processes, produces near-minimal solutions.

#### 3.1. Minimal set covering (the MSC problem)

The Minimal Set Cover (MSC) problem is a classical question in computer science and complexity theory [57], [58]. It is a problem “whose study has led to the development of fundamental techniques for the entire field” of approximation algorithms [59], and it was one of Karp’s 21 core NP-complete problems shown to be NP-complete in his landmark 1972 paper [60]. It can be informally described as follows. Given a collection of 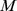 sets 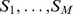, each is a subset of the integers 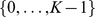, what is the minimum number of sets whose union contains all the integers 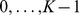
? For example, if the collection of sets (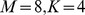) is: 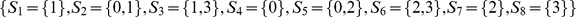. Note that sets 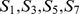 together *cover* the elements, that is, 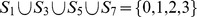. However, it is a cover of size 4. On the other hand, 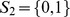 and 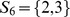 also form a cover: 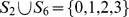. No single set contains all the elements, and thus the minimal set cover is of size 2.

Next, we explain how an MSC problem can be expressed in Boolean form, using the example above. Consider the Boolean equations.
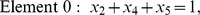


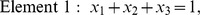


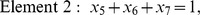


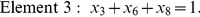
(23)


Here, the variable 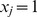 if set 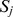 is part of a set cover. Thus, the first line above says that at least one of the sets 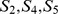 needs to be in the cover in order for element 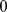 to be included. Since all elements need to be included, a solution to the CNF expression.
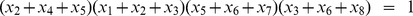
results in a cover. For a minimal cover, we want as few variables 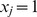 as possible.

#### 3.2. The minimal-network problem is at least as hard as the MSC problem

In the standard approach [58], to demonstrate that problem 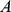 is hard, one shows that a known hard problem 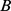 can be transformed into 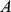. In this manner, a putative fast (polynomial-time) algorithm for 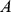 must also be able to solve 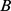 quickly and since 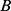 is known to be hard, therefore 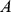 must be hard. The transformation from 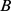 to 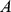 needs to occur in polynomial-time so that the entire process takes polynomial-time.

We now show how an arbitrary instance of an MSC problem can be transformed into an equivalent minimal-network problem in polynomial time so that a putative fast algorithm for the minimal network problem would have to solve the MSC problem in polynomial-time. The transformation is best explained via an example – we use the example MSC problem above in Eq. (23). We need to find a feasible Boolean biological process whose equations match Eq. (23).

**Figure 1:**
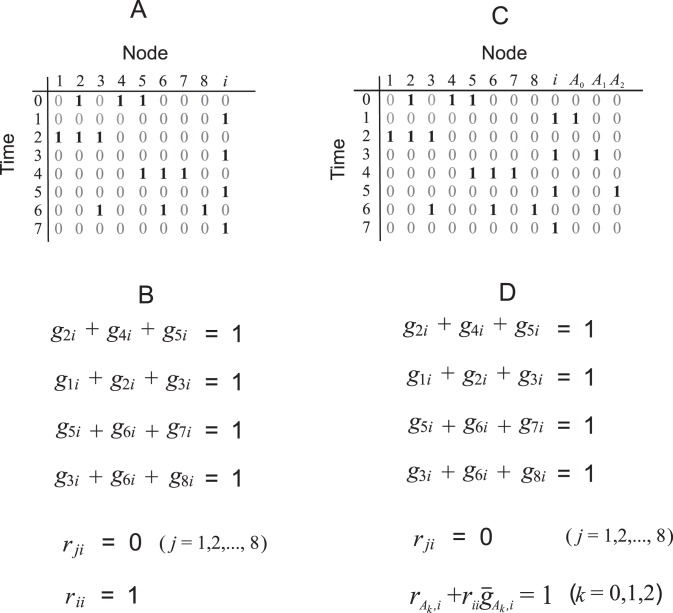
The transformation of a minimal set covering problem into the problem of finding a minimal biological network for a given Boolean process. (A) A Boolean process which does not always have a network solution. (B) The Boolean equations for network edges incoming to node 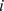, according to the process in (A). (C) A Boolean process that is guaranteed to have a network solution. (D) The Boolean equations for network edges incoming to node 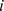, according to the process in (C).

Figure 1(A) shows a process with 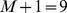 nodes and 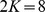 time steps 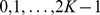. The 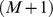-th column is for a molecule called 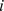, whose transitions will define the desired Boolean process. At time 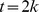 (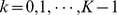), we set 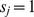 for those 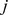 appearing in the equation for element 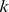. For example, the equation for set element 3 is 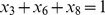; we therefore set 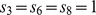 in the row 6 of the process. As for node 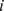, we set 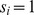 at rows 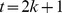 (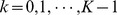).

Next, consider the Boolean equations for node 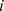, shown in Fig. 1(B). The first four equations are exactly Eq. (23) after replacing 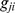 with 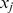. Notice that in the column for 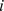, whenever node 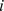 makes a 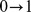 transition, the other nodes in the row that are “on” cannot have an inhibitory edge. Thus, 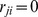 for these nodes. Similarly, the only way in which the 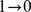 transition can occur is due to self-degradation, i.e., 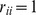.

At first, it appears that this transformation is sufficient. However, notice that nodes 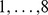 need to make a 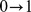 transition in alternate rows. Unfortunately, the transitions rely solely on the activations from node 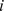, which would result in conflicts. Figure 1(A) indicates that node 3 makes a 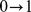 transition at 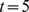, and therefore there must be a stimulatory edge from node 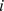 to node 3 (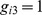). Therefore, this stimulatory edge must have activated node 3 at time 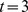 (i.e., 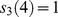), which contradicts the Boolean process and which shows 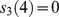.

Based on the process in Fig. 1(A), we construct a new process in Fig. 1(C) whose feasibility is guaranteed. The process has 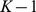 additional nodes, denoted by 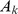 (for 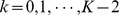). The node 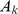 is responsible solely for all the activations at time 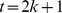. File S1 E explains why the resulting process is always feasible.

If the minimal network problem for the Boolean process in Fig. 1(C) were solved in polynomial-time, then the equations in Fig. 1(D) and consequentially Eq. (23) could be solved in polynomial-time. This would imply that the MSC problem can be solved in polynomial-time and that the NP  =  P problem is solved in the affirmative. Therefore, it is unlikely that there is a polynomial-time algorithm to find a minimal network.

#### Theorem

Given a Boolean process, the problem of finding a minimal network (a network with the smallest number of edges) to realize it, based on the model Eq. (2), is NP-complete.

#### 3.3. A heuristic algorithm for finding an approximated minimal network

While the association with the cover problem proved that the problem is hard, it also suggests a heuristic. It is known that a simple “greedy” algorithm for the set-cover problem is effective in practice and in fact can be shown to result in covers within 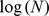 of optimal [61]. The greedy algorithm for set-cover works as follows: first select the set that covers the most elements. Then, mark the covered elements as “covered.” Then, select the set that covers the most uncovered elements, and repeat in this fashion. The greedy algorithm was used in various biological fields including the inference of sparse Boolean functions [56], [62]–[64].

For our minimal network problem, we first apply the greedy algorithm to only the equations containing stimulatory edges, which approximately identifies a set of minimal stimulatory edges. The results simplify the remaining equations, which contain only inhibitory edges. By applying the greedy algorithm again, an approximated minimal network is found. We consider separately the two cases of 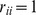 and 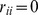. The algorithm is described as follows.

1. // Start with the first node
  - *i ←* 1
2. // The case *r_ii_*  =  1
  - *r_ii_ ←* 1
  - Use the greedy algorithm to find a set of stimulatory edges
  - Use the greedy algorithm to find a set of inhibitory edges
3. // The case *r_ii_*  =  0
  - *r_ii_ ←* 0
  - Use the greedy algorithm to find a set of stimulatory edges
  - Use the greedy algorithm to find a set of inhibitory edges
4. Choose the smaller edge set from steps 2 and 3 above for node *i*
5. // Move onto the next node
  - *i ← i* + 1
  - if *i > N* goto 6
  - *else goto 2*
  - *endif*
6. Stop

#### 3.4. Validation of the algorithm for finding an approximated minimal network

**Figure 2:**
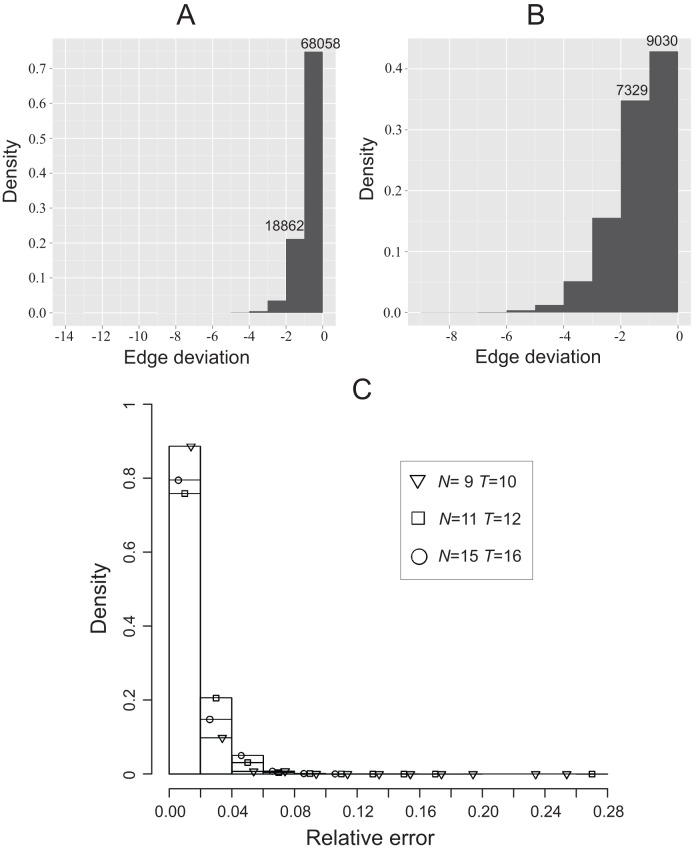
Validation of a heuristic algorithm for finding an approximated minimal network. A large number of feasible Boolean processes were generated. For each process, an approximated minimal network was determined by the heuristic algorithm; the actual minimal network is also computed by time-consuming enumeration. The histogram shows how the number of processes spread over the deviation of estimation (actual minimality minus estimated minimality). (A) 90,635 processes with 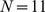 and 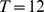. (B) 21,000 processes with 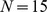 and 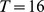. (C) Processes with 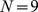 and 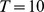 as compared with other processes.

To test the efficacy of the greedy heuristic, we constructed 90,635 feasible processes, each with 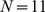 and 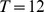. For each of these 90,635 processes, we used the algorithm to seek an approximated minimal network. As a comparison, we also compute the actual minimal networks, by time-consuming brute force enumerations, whereby we can assess how far off from optimal the heuristic’s solutions are. The results are shown in Fig. 2(A). One sees that the approximated minimal network for each of the 68,058 processes (75.1% of total) is a genuine minimal network. The approximated minimal network for each of 18,862 processes (20.8% of total) has only one more edge than the genuine minimal network. Only 4% processes have two or more edges than minimal.

We also constructed 21,000 feasible processes each with 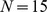 and 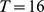. The results for these are shown in Fig. 2(B). One sees that the approximated minimal network for each of the 9030 processes (43% of total) is a genuine minimal network. The approximated minimal network for each of the 7329 processes (34.9% of total) has one more edge than the genuine minimal network. Only 22% processes give deviations more than one edge. We also studied many 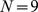 and 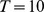 processes, with the results presenting in Fig. 2(C), together with the other results.

The above numerical results indicate that the heuristic algorithm is very accurate, with high confidence for at most one additional edge than optimal.

File S1 F and G further provide examples of finding minimal networks.

### 4. Designability: How Many Solutions?

We consider one more computational aspect of Boolean networks: computing the *designability* of a biological process. The designability of a process is the number of network solutions, that is, the total number of networks that can produce a given process. A process with high designability is likely to be more robust and therefore survive natural selection. If a biological process has only one network solution, for example, then it is unlikely that random mutation would find that network. In other words, processes with high designability are more likely to occur (or be discovered by evolution) than low-designability ones [5], [65].

Unfortunately, computing the designability of a process appears to take exponential time, a result that is not unexpected because the problem is similar to many such combinatorial enumeration problems [66]. Instead, we seek a fast approximate algorithm. Our approach is to use a type of logistic regression on certain features of a problem. These features are obtained during the simplification procedure described earlier in the feasibility solution.

During simplification, it is easy to obtain the following: 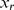, the number of inhibitory edges (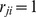); 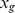, the number of stimulatory edges (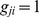); 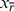, the number of edges that cannot be inhibitory (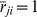); 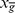, the number of edges that cannot be stimulatory (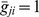); 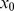, the number of nodes that cannot connect to node 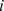 (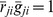); 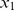, the number of nodes that can be associated with node 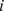 in arbitrary way (inhibitory, stimulatory, or “no connection”).

We now assume that the approximated log-designability (denoted by 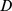) is a linear function of 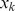:
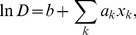
(24)where 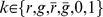, 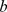 and 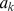 are unknown coefficients. The coefficients can be estimated by minimizing the difference between the approximated designability 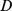 and the exact designability 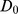 for some test cases. To this end, we generate 800,000 random Boolean process, each of which has 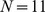 and 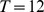. For each process, we calculate the exact designability 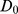 and the values of 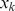, for 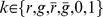. We then perform a least-square data fitting to obtain the values of the coefficients. The results are: 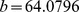, 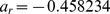, 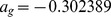, 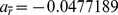, 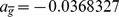, 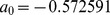, and 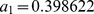.

**Figure 3:**
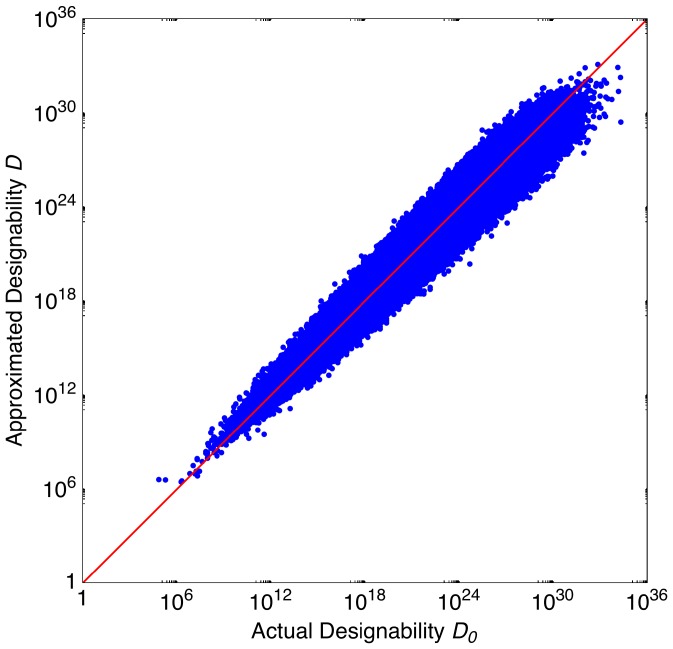
Designability approximation. The 800,000 dots in the picture each represent a random Boolean process. For each dot, the actual designability 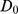 and the approximated designability 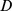 are represented by the 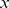 axis value and the 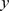 axis value, respectively. The diagonal corresponds to the ideal fitting.

In Fig. (3), each dot represents one Boolean process; the 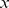 and 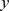 axes represent 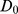 and 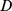, respectively. One sees that the fitting is successful. The dots locate along the diagonal with only small deviations. Therefore, we now have an empirical formula to estimate designability with sensible accuracy. Although it is not very accurate to assume *N*- and *T*- independent regression coefficients and use them to estimate designability, the estimation can still be very useful because it becomes very difficult to compute the exact designability for large *N* and *T*.

## Discussion

Biological systems are generally large, involving many complicated molecular interactions among numerous working components. In this case, a “coarse grained” description such as a Boolean network model is often a useful step towards understanding a system. Furthermore, a study of general Boolean networks may help elucidate network design principles in biological systems. The Boolean framework in this paper results in a single analytical equation (Eq. 2) to integrate the information about network structure (edge variables) and biological process (state variables). This has rendered the structure-function relationship tractable. When structural information is known, one can use Eq. (2) to study the dynamics and learn the biological function. When the process is known, one can use Eq. (2) to characterize the network space constrained by the process. When both information are partially known, Eq. (2) can still help enumerate the structure-function combinations.

In this paper, we have answered some key questions about the computational complexity of the network inference problem in Boolean networks that feature dominant inhibition. The first is the feasibility problem: is the solution space null? The second is the minimal network problem: what are the fundamental building blocks of the space, namely those networks with the least number of edges? The third is the designability problem: how big is the solution space if it is not null?

Fast algorithms provide the benefit of being able to study many types of processes and to explore the statistics of processes. For example, one is interested in what general features of a process make a process a biological one suited to evolution. Is it the case, for example, that a small minimal network acts as the core with additional edges accumulated during evolution, and does a large multiplicity help maintain stability against mutation?

Beyond a rigorous classification of problem complexity, the present study also offers accurate heuristic algorithms that run in polynomial time. This will be crucial for handling both large-scale datasets and the inevitable statistical noise. The former requires a well-controlled scaling as observed in these polynomial heuristics and the latter requires iterative applications of these algorithms for noise sampling on a trial-and-error basis.

## Supporting Information

File S1 **A collection of technical details.**
(PDF)

## References

[1] Bornholdt S (2005). Less is more in modeling large genetic networks.. Science.

[2] Kauffman SA (1993). The Origins of Order: Self-Organization and Selection in Evolution.. Oxford: Oxford University Press.

[3] Li F, Long T, Lu Y, Ouyang Q, Tang C (2004). The yeast cell-cycle network is robustly designed.. Proc Natl Acad Sci USA.

[4] Lau K, Ganguli S, Tang C (2007). Function constrains network architecture and dynamics: A case study on the yeast cell cycle Boolean network.. Phys Rev E.

[5] Nochomovitz YD, Li H (2006). Highly designable phenotypes and mutational buffers emerge from a systematic mapping between network topology and dynamic output.. Proc Natl Acad Sci USA.

[6] Kashtan N, Alon U (2005). Spontaneous evolution of modularity and network motifs.. Proc Natl Acad Sci USA.

[7] Emmert-Streib F (2010). A brief introduction to complex networks and their analysis.. In: Dehmer M, editor, Structural Analysis of Complex Networks, New York: Birkhäuser..

[8] Estrada E (2011). The Structure of Complex Networks: Theory and Applications.. Oxford: Oxford University Press.

[9] Erdös P, Rényi A (1959). On random graphs.. Publicationes Mathematicae.

[10] Watts DJ, Strogatz SH (1998). Collective dynamics of small-world networks.. Nature.

[11] Barabasi AL, Albert R (1999). Emergence of scaling in random networks.. Science.

[12] Bornholdt S, Schuster HG (2003). Handbook of graphs and networks: from the genome to the internet.. New York: Wiley.

[13] Emmert-Streib F, Dehmer M (2007). Information theoretic measures of UHG graphs with low computational complexity.. Appl Math Comput.

[14] Dehmer M, Emmert-Streib F (2008). Structural information content of networks: graph entropy based on local vertex functionals.. Comput Biol Chem.

[15] Dehmer M, Borgert S, Emmert-Streib F (2008). Entropy bounds for hierarchical molecular networks.. PLoS ONE.

[16] Dehmer M, Varmuza K, Borgert S, Emmert-Streib F (2009). On entropy-based molecular descriptors: Statistical analysis of real and synthetic chemical structures.. J Chem Inf Model.

[17] Milo R, Shen-Orr S, Itzkovitz S, Kashtan N, Chklovskii D (2002). Network motifs: simple building blocks of complex networks.. Science.

[18] Jeong H, Tombor B, Albert R, Oltvai ZN, Barabási AL (2000). The large-scale organization of metabolic networks.. Nature.

[19] Greenbury SF, Johnston IG, Smith MA, Doye JP, Louis AA (2010). The effect of scale-free topology on the robustness and evolvability of genetic regulatory networks.. J Theor Biol.

[20] Bansal M, Belcastro V, Ambesi-Impiombato A, di Bernardo D (2007). How to infer gene networks from expression profiles.. Mol Syst Biol.

[21] D’haeseleer P, Liang S, Somogyi R (2000). Genetic network inference: from co-expression clustering to reverse engineering.. Bioinformatics.

[22] Shmulevich I, Zhang W (2002). Binary analysis and optimization-based normalization of gene expression data.. Bioinformatics.

[23] Needham CJ, Manfield IW, Bulpitt AJ, Gilmartin PM, Westhead DR (2009). From gene expression to gene regulatory networks in arabidopsis thaliana.. BMC Syst Biol.

[24] Altay G, Emmert-Streib F (2011). Structural influence of gene networks on their inference: analysis of C3NET.. Biol Direct.

[25] Ideker T, Thorsson V, Karp R (2000). Discovery of regulatory interactions through perturbation: inference and experimental design.. In: Pacific Symposium on Biocomputing. World Scientific Maui, Hawaii, volume 5, 302–313..

[26] Akutsu T, Kuhara S, Maruyama O, Miyano S (2003). Identification of genetic networks by strategic gene disruptions and gene overexpressions under a Boolean model.. Theor Comput Sci.

[27] Perkins T, Hallett M (2010). A trade-off between sample complexity and computational complexity in learning Boolean networks from time-series data.. IEEE/ACM Trans Comput Biol Bioinform.

[28] Saithong T, Bumee S, Liamwirat C, Meechai A (2012). Analysis and practical guideline of constraint-based Boolean method in genetic network inference.. PLoS ONE.

[29] Wang G, Du C, Simha R, Rong Y, Xiao Y (2010). Process-based network decomposition reveals backbone motif structure.. Proc Natl Acad Sci U S A.

[30] Albert R, Othmer HG (2003). The topology of the regulatory interactions predicts the expression pattern of the segment polarity genes in Drosophila melanogaster.. J Theor Biol.

[31] Kauffman S (1969). Metabolic stability and epigenesis in randomly constructed genetic nets.. J Theor Biol.

[32] Glass L, Kauffman S (1973). The logical analysis of continuous, non-linear biochemical control networks.. J Theor Biol.

[33] Tan N, Ouyang Q (2006). Design of a network with state stability.. J Theor Biol.

[34] Fortuna MA, Melián CJ (2007). Do scale-free regulatory networks allow more expression than random ones?. J Theor Biol.

[35] Chandru V, Coullard CR, Hammer PL, Montanez M, Sun X (1990). On renamable Horn and generalized Horn functions.. Ann Math Artif Intell.

[36] Dowling WF, Gallier JH (1984). Linear time algorithms for testing the satisfiability of propositional Horn formulae.. J Logic Program.

[37] Alon U (2003). Biological networks: the tinkerer as an engineer.. Science.

[38] Mendelson E (1997). Introduction to Mathematical Logic.. London: Chapman and Hall.

[39] Brassard G, Bratley P (1996). Fundamentals of Algorithmics.. Prentice Hall.

[40] Cook SA (1971). The complexity of theorem-proving procedures.. In: Proceedings of the third annual ACM symposium on Theory of computing. ACM, 151–158..

[41] Aspvall B, Plass MF, Tarjan RE (1979). A linear-time algorithm for testing the truth of certain quantified Boolean formulas.. Inform Process Lett.

[42] Horn A (1951). On sentences which are true of direct unions of algebras.. J Symb Logic.

[43] Chang CC, Morel AC (1958). On closure under direct product.. J Symb Logic.

[44] Keisler HJ (1965). Reduced products and Horn classes.. Trans Amer Math Soc.

[45] Cook S, Nguyen P (2010). Logical foundations of proof complexity.. Cambridge: Cambridge University Press.

[46] Perkins TJ, Wilds R, Glass L (2010). Robust dynamics in minimal hybrid models of genetic networks.. Phil Trans R Soc A.

[47] Finlayson MR, Helfer-Hungerbühler AK, Philippsen P (2011). Regulation of exit from mitosis in multinucleate ashbya gossypii cells relies on a minimal network of genes.. Mol Biol Cell.

[48] Howes R, Eccleston L, Gonalves J, Stan GB, Warnick S (1998). Dynamical structure analysis of sparsity and minimality heuristics for reconstruction of biochemical networks.. In: Proceedings of the 47th IEEE Conference on Decision and Control. IEEE, Cancun, Mexico: IEEE..

[49] Raychaudhuri S (2010). A minimal model of signaling network elucidates cell-to-cell stochastic variability in apoptosis.. PLoS ONE.

[50] Okazaki N, Asano R, Kinoshita T, Chuman H (2008). Simple computational models of type I/type II cells in Fas signaling-induced apoptosis.. J Theor Biol.

[51] van Beek P, Dechter R (1995). On the minimality and global consistency of row-convex constraint networks.. J ACM.

[52] Blum A, Langley P (1997). Selection of relevant features and examples in machine learning.. Artif Intell.

[53] Mossel E, O’Donnell R, Servedio RP (2003). Learning juntas.. In: Proceedings of the thirty-fifth annual ACM symposium on Theory of computing. ACM, 206–212..

[54] Mukherjee S, Pelech S, Neve RM, Kuo WL, Ziyad S (2009). Sparse combinatorial inference with an application in cancer biology.. Bioinformatics.

[55] Hogan JM, Diederich J (2001). Recruitment learning of Boolean functions in sparse random networks.. Int J Neural Syst.

[56] Fukagawa D, Akutsu T (2005). Performance analysis of a greedy algorithm for inferring Boolean functions.. Inform Process Lett.

[57] Chvatal V (1979). A greedy heuristic for the set-covering problem.. Math Oper Res.

[58] Garey MR, Johnson DS (1979). Computers and Intractability: A Guide to the Theory of NP-Completeness.. New York: W.H. Freeman.

[59] Alon N, Moshkovitz D, Safra S (2006). Algorithmic construction of sets for k-restrictions.. ACM Trans Algorithms.

[60] Karp RM (1972). Reducibility among combinatorial problems.. In: Miller RE, Thatcher JW, editors, Complexity of Computer Computations, New York: Plenum..

[61] Chvatal V (1979). A greedy heuristic for the set covering problem.. Math Oper Res 4.

[62] Kosaraju SR, Schäffer AA, Biesecker LG (1998). Approximation algorithms for a genetic diagnostics problem.. J Comput Biol.

[63] Doi K, Imai H (1997). Greedy algorithms for finding a small set of primers satisfying cover and length resolution conditions in PCR experiments.. Genome Inform Ser Workshop Genome Inform.

[64] Chowdhury SA, Koyuẗurk M (2010). Identification of coordinately dysregulated subnetworks in complex phenotypes.. Pac Symp Biocomput.

[65] Isalan M, Lemerle C, Michalodimitrakis K, Horn C, Beltrao P (2008). Evolvability and hierarchy in rewired bacterial gene networks.. Nature.

[66] Vassilevska V, Williams R (2009). Finding, minimizing, and counting weighted subgraphs.. 41st ACM Symposium on Theory of Computing.

