# Supplementary material for: Process-Driven Inference of Biological Network Structure: Feasibility, Minimality, and Multiplicity
Source: PLoS One. 2012 Jul 18;7(7):e40330. doi: 10.1371/journal.pone.0040330 (PMC3399897; doi:10.1371/journal.pone.0040330)
Supplement: File S1 — A collection of technical details. (PDF) [file pone.0040330.s001.pdf]

Supporting Information

A. Substantiation of Eq. (2) according to the four type of state transitions

Equation (2) is in a compact form, which might be inconvenient for various applications. By defining $\theta_t(i) = \{\ j\ \ |s_j\,(t) = 1;\ \ j \neq i\}$, the set of active nodes other than i at time t, Eq. (2) is re-written into four equations each correspond to a type of state transition of node i:

$$0 \to 1: \qquad \sum_{j\in\theta_t(i)} g_{ji} \cdot \prod_{j\in\theta_t(i)} \overline{r}_{ji} = 1, \tag{S.1}$$

$$1 \to 1: \qquad \left(\sum_{j\in\theta_t(i)} g_{ji} + \bar{r}_{ii}\right) \cdot \prod_{j\in\theta_t(i)} \overline{r}_{ji} = 1, \tag{S.2}$$

$$0 \to 0: \qquad \sum_{j\in\theta_t(i)} g_{ji} \cdot \prod_{j\in\theta_t(i)} \overline{r}_{ji} = 0, \tag{S.3}$$

$$1 \to 0: \qquad \left(\sum_{j\in\theta_t(i)} g_{ji} + \bar{r}_{ii}\right) \cdot \prod_{j\in\theta_t(i)} \overline{r}_{ji} = 0. \tag{S.4}$$

As a simplification, we perform complementation on Eqs. (S.3) and (S.4) so that the right hand sides of all the equations are 1.

$$0 \to 1: \qquad \sum_{j\in\theta_t(i)} g_{ji} \cdot \prod_{j\in\theta_t(i)} \overline{r}_{ji} = 1, \tag{S.5}$$

$$1 \to 1: \qquad \left(\sum_{j\in\theta_t(i)} g_{ji} + \bar{r}_{ii}\right) \cdot \prod_{j\in\theta_t(i)} \overline{r}_{ji} = 1, \tag{S.6}$$

$$0 \to 0: \qquad \prod_{j\in\theta_t(i)} \overline{g}_{ji} + \sum_{j\in\theta_t(i)} r_{ji} = 1, \tag{S.7}$$

$$1 \to 0: \qquad r_{ii} \prod_{j\in\theta_t(i)} \overline{g}_{ji} + \sum_{j\in\theta_t(i)} r_{ji} = 1. \tag{S.8}$$

From Eqs. (S.5) and (S.6) one immediately finds $r_{ji} = 0$ for some nodes j. By sweeping all the equations ($t = 0, 1, \cdots, T-1$), a set $\Theta = \{\ j\ \ |r_{ji} = 0;\ \ j \neq i\}$ is formed to collect all such nodes. Equations (S.7) and (S.8) can be further simplified by substituting $r_{ji} = 0$ (for

$j \in \Theta$) into them:

$$0 \to 1: \qquad \sum_{j\in\theta_t(i)} g_{ji} = 1, \tag{S.9}$$

$$1 \to 1: \qquad \sum_{j\in\theta_t(i)} g_{ji} + \bar{r}_{ii} = 1, \tag{S.10}$$

$$0 \to 0: \qquad \prod_{j\in\theta_t(i)} \overline{g}_{ji} + \sum_{j\in\theta_t(i)\backslash\Theta} r_{ji} = 1, \tag{S.11}$$

$$1 \to 0: \qquad r_{ii} \prod_{j\in\theta_t(i)} \overline{g}_{ji} + \sum_{j\in\theta_t(i)\backslash\Theta} r_{ji} = 1, \tag{S.12}$$

where the set $\theta_t(i)\backslash\Theta$ contains the nodes in $\theta_t(i)$ but with the ones in Θ excluded.

B. Horn formula representation of Boolean equations for a small process

Table S1: A small Boolean process with $N = 4$ nodes and $T = 4$ time steps.

Time	s_1	s_2	s_3	s_4
0	1	1	0	0
1	1	0	1	0
2	0	0	0	1
3	0	0	0	1

A Boolean process for $N = 4$ nodes and $T = 4$ time steps is given in Table S1. We focus on node $i = 4$ and outline the simplification procedure and the conversion to Horn-clause form in four steps below.

B.1. Boolean equations

$$\begin{aligned}\overline{g}_{1i}\overline{g}_{2i} + r_{1i} + r_{2i} &= 1\\ (g_{1i} + g_{3i})\,\overline{r}_{1i}\overline{r}_{3i} &= 1\\ \overline{r}_{ii} &= 1\end{aligned}$$

B.2. Simplified equations

$$r_{1i} = r_{3i} = r_{ii} = 0$$

and

$$\begin{aligned}\overline{g}_{1i}\overline{g}_{2i} + r_{2i} &= 1\\ g_{1i} + g_{3i} &= 1\end{aligned}$$

B.3. CNF for the simplified equations

$$(\overline{g}_{1i} + r_{2i})\,(\overline{g}_{2i} + r_{2i})\,(g_{1i} + g_{3i}) = 1 \tag{S.13}$$

B.4. Horn formula

From the above expression one sees that nodes 1 and 3 belong to the set $\Theta = \{a, b, c, \cdots\}$, node 2 belongs to the set $\{A, B, C, \cdots\}$, thus $\overline{g}_{1i} = G_{1i}$ $(g_{1i} = \overline{G}_{1i})$, $g_{3i} = \overline{G}_{3i}$, and $r_{2i} = \overline{R}_{2i}$ as described in Section 3.3. The Horn formula of the above CNF is

$$\left(G_{1i} + \overline{R}_{2i}\right)\left(\overline{g}_{2i} + \overline{R}_{2i}\right)\left(\overline{G}_{1i} + \overline{G}_{3i}\right) = 1. \tag{S.14}$$

C. Horn formula representation of Boolean equations for a large process

Table S2: A Boolean process with $N = 22$ nodes and $T = 15$ time steps.

Time	s_1	s_2	s_3	s_4	s_5	s_6	s_7	s_8	s_9	s_{10}	s_{11}	s_{12}	s_{13}	s_{14}	s_{15}	s_{16}	s_{17}	s_{18}	s_{19}	s_{20}	s_{21}	s_{22}
0	0	0	1	1	1	1	0	1	1	0	1	1	1	1	1	1	1	0	0	1	0	0
1	0	0	0	0	0	0	0	0	0	0	0	0	0	0	0	0	0	1	0	0	1	0
2	0	1	0	0	0	0	1	0	0	1	0	0	0	0	0	0	0	1	0	1	1	0
3	0	0	0	0	1	0	0	1	0	0	0	0	0	0	0	0	0	0	0	1	0	0
4	0	0	0	1	1	0	0	1	1	1	0	1	1	0	0	0	1	0	0	0	1	1
5	1	0	0	0	1	0	0	0	0	0	0	0	1	0	0	0	0	1	0	0	0	0
6	0	0	0	0	1	0	0	1	0	0	0	1	0	1	0	0	0	0	1	0	1	1
7	0	0	0	1	0	0	0	0	0	0	0	0	0	0	1	0	0	1	0	0	1	0
8	0	1	1	0	0	0	1	0	0	0	0	0	0	0	0	0	0	1	0	0	1	0
9	0	0	0	0	1	0	1	1	0	0	0	1	0	0	0	0	0	0	0	1	0	0
10	0	0	0	0	1	0	0	1	0	0	0	1	1	0	0	0	0	0	0	0	0	1
11	1	0	0	0	1	1	0	1	1	0	0	1	1	0	1	0	0	1	0	0	1	1
12	0	0	0	0	0	0	0	0	0	0	0	0	0	0	0	0	0	0	0	0	1	0
13	0	1	0	0	0	0	0	0	1	0	0	0	1	0	1	0	0	1	0	1	1	0
14	0	0	0	0	0	0	0	0	0	0	0	0	0	0	0	0	0	0	0	0	1	0

A larger Boolean process than the previous example has $N = 22$ nodes and $T = 15$ time steps as shown in Table S2. We focus on node $i = 5$. Similarly, we work out the simplification procedure and the conversion to Horn-clause form in four steps below.

C.1. Boolean equations

$$
\begin{aligned}
r_{ii}\overline{g}_{3i}\overline{g}_{4i}\overline{g}_{6i}\overline{g}_{8i}\overline{g}_{9i}\overline{g}_{11,i}\overline{g}_{12,i}\overline{g}_{13,i}\overline{g}_{14,i}\overline{g}_{15,i}\overline{g}_{16,i}\overline{g}_{17,i}\overline{g}_{20,i} + r_{3i} + r_{4i} + r_{6i} \; + & \\
r_{8i} + r_{9i} + r_{11,i} + r_{12,i} + r_{13,i} + r_{14,i} + r_{15,i} + r_{16,i} + r_{17,i} + r_{20,i} &= 1 \\
\overline{g}_{18,i}\overline{g}_{21,i} + r_{18,i} + r_{21,i} &= 1 \\
\overline{r}_{2i}\overline{r}_{7i}\overline{r}_{10,i}\overline{r}_{18,i}\overline{r}_{20,i}\overline{r}_{21,i}\,(g_{2i} + g_{7i} + g_{10,i} + g_{18,i} + g_{20,i} + g_{21,i}) &= 1 \\
\overline{r}_{8i}\overline{r}_{20,i}(\overline{r}_{ii} + g_{8i} + g_{20,i}) &= 1 \\
\overline{r}_{4i}\overline{r}_{8i}\overline{r}_{9i}\overline{r}_{10,i}\overline{r}_{12,i}\overline{r}_{13,i}\overline{r}_{17,i}\overline{r}_{21,i}\overline{r}_{22,i} & \\
(\overline{r}_{ii} + g_{4i} + g_{8i} + g_{9i} + g_{10,i} + g_{12,i} + g_{13,i} + g_{17,i} + g_{21,i} + g_{22,i}) &= 1 \\
\overline{r}_{1i}\overline{r}_{13,i}\overline{r}_{18,i}(\overline{r}_{ii} + g_{1i} + g_{13,i} + g_{18,i}) &= 1 \\
r_{ii}\overline{g}_{8i}\overline{g}_{12,i}\overline{g}_{14,i}\overline{g}_{19,i}\overline{g}_{21,i}\overline{g}_{22,i} + r_{8i} + r_{12,i} + r_{14,i} + r_{19,i} + r_{21,i} + r_{22,i} &= 1 \\
\overline{g}_{4i}\overline{g}_{15,i}\overline{g}_{18,i}\overline{g}_{21,i} + r_{4i} + r_{15,i} + r_{18,i} + r_{21,i} &= 1 \\
\overline{r}_{2i}\overline{r}_{3i}\overline{r}_{7i}\overline{r}_{18,i}\overline{r}_{21,i}\,(g_{2i} + g_{3i} + g_{7i} + g_{18,i} + g_{21,i}) &= 1 \\
\overline{r}_{7i}\overline{r}_{8i}\overline{r}_{12,i}\overline{r}_{20,i}(\overline{r}_{ii} + g_{7i} + g_{8i} + g_{12,i} + g_{20,i}) &= 1 \\
\overline{r}_{8i}\overline{r}_{12,i}\overline{r}_{13,i}\overline{r}_{22,i}(\overline{r}_{ii} + g_{8i} + g_{12,i} + g_{13,i} + g_{22,i}) &= 1 \\
r_{ii}\overline{g}_{1i}\overline{g}_{6i}\overline{g}_{8i}\overline{g}_{9i}\overline{g}_{12,i}\overline{g}_{13,i}\overline{g}_{15,i}\overline{g}_{18,i}\overline{g}_{21,i}\overline{g}_{22,i} + r_{1i} + r_{6i} \; + & \\
r_{8i} + r_{9i} + r_{12,i} + r_{13,i} + r_{15,i} + r_{18,i} + r_{21,i} + r_{22,i} &= 1 \\
\overline{g}_{21,i} + r_{21,i} &= 1 \\
\overline{g}_{2i}\overline{g}_{9i}\overline{g}_{13,i}\overline{g}_{15,i}\overline{g}_{18,i}\overline{g}_{20,i}\overline{g}_{21,i} + r_{2i} + r_{9i} + r_{13,i} + r_{15,i} + r_{18,i} + r_{20,i} + r_{21,i} &= 1. \qquad \text{(S.15)}
\end{aligned}
$$

C.2. Simplified equations

$$
\begin{aligned}
r_{1i} = r_{2i} = r_{3i} = r_{4i} = r_{7i} = r_{8i} = r_{9i} = r_{10,i} = r_{12,i} &= \\
r_{13,i} = r_{17,i} = r_{18,i} = r_{20,i} = r_{21,i} = r_{22,i} = g_{18,i} = g_{21,i} &= 0
\end{aligned}
$$

and

$$
\begin{aligned}
r_{ii}\overline{g}_{3i}\overline{g}_{4i}\overline{g}_{6i}\overline{g}_{8i}\overline{g}_{9i}\overline{g}_{11,i}\overline{g}_{12,i}\overline{g}_{13,i}\overline{g}_{14,i}\overline{g}_{15,i}\overline{g}_{16,i}\overline{g}_{17,i}\overline{g}_{20,i} & \\
+r_{6i} + r_{11,i} + r_{14,i} + r_{15,i} + r_{16,i} &= 1 \\
g_{2i} + g_{7i} + g_{10,i} + g_{20,i} &= 1 \\
\overline{r}_{ii} + g_{8i} + g_{20,i} &= 1 \\
\overline{r}_{ii} + g_{4i} + g_{8i} + g_{9i} + g_{10,i} + g_{12,i} + g_{13,i} + g_{17,i} + g_{22,i} &= 1 \\
\overline{r}_{ii} + g_{1i} + g_{13,i} &= 1 \\
r_{ii}\overline{g}_{8i}\overline{g}_{12,i}\overline{g}_{14,i}\overline{g}_{19,i}\overline{g}_{22,i} + r_{14,i} + r_{19,i} &= 1 \\
\overline{g}_{4i}\overline{g}_{15,i} + r_{15,i} &= 1 \\
g_{2i} + g_{3i} + g_{7i} &= 1 \\
\overline{r}_{ii} + g_{7i} + g_{8i} + g_{12,i} + g_{20,i} &= 1 \\
\overline{r}_{ii} + g_{8i} + g_{12,i} + g_{13,i} + g_{22,i} &= 1 \\
r_{ii}\overline{g}_{1i}\overline{g}_{6i}\overline{g}_{8i}\overline{g}_{9i}\overline{g}_{12,i}\overline{g}_{13,i}\overline{g}_{15,i}\overline{g}_{22,i} + r_{6i} + r_{15,i} &= 1 \\
\overline{g}_{2i}\overline{g}_{9i}\overline{g}_{13,i}\overline{g}_{15,i}\overline{g}_{20,i} + r_{15,i} &= 1. \qquad \text{(S.16)}
\end{aligned}
$$

C.3. CNF for the left hand of the simplified equations

$$
\begin{aligned}
&(r_{ii} + r_{6i} + r_{11,i} + r_{14,i} + r_{15,i} + r_{16,i}) (\overline{g}_{3i} + r_{6i} + r_{11,i} + r_{14,i} + r_{15,i} + r_{16,i}) \\
&(\overline{g}_{4i} + r_{6i} + r_{11,i} + r_{14,i} + r_{15,i} + r_{16,i}) (\overline{g}_{6i} + r_{11,i} + r_{14,i} + r_{15,i} + r_{16,i}) \\
&(\overline{g}_{8i} + r_{6i} + r_{11,i} + r_{14,i} + r_{15,i} + r_{16,i}) (\overline{g}_{9i} + r_{6i} + r_{11,i} + r_{14,i} + r_{15,i} + r_{16,i}) \\
&\left(\overline{g}_{11,i} + r_{6i} + r_{14,i} + r_{15,i} + r_{16,i}\right) \left(\overline{g}_{12,i} + r_{6i} + r_{11,i} + r_{14,i} + r_{15,i} + r_{16,i}\right) \\
&\left(\overline{g}_{13,i} + r_{6i} + r_{11,i} + r_{14,i} + r_{15,i} + r_{16,i}\right) \left(\overline{g}_{14,i} + r_{6i} + r_{11,i} + r_{15,i} + r_{16,i}\right) \\
&\left(\overline{g}_{15,i} + r_{6i} + r_{11,i} + r_{14,i} + r_{16,i}\right) \left(\overline{g}_{16,i} + r_{6i} + r_{11,i} + r_{14,i} + r_{15,i}\right) \\
&\left(\overline{g}_{17,i} + r_{6i} + r_{11,i} + r_{14,i} + r_{15,i} + r_{16,i}\right) \left(\overline{g}_{20,i} + r_{6i} + r_{11,i} + r_{14,i} + r_{15,i} + r_{16,i}\right) \\
&(g_{2i} + g_{7i} + g_{10,i} + g_{20,i}) (\overline{r}_{ii} + g_{8i} + g_{20,i}) (\overline{r}_{ii} + g_{1i} + g_{13,i}) \\
&(\overline{r}_{ii} + g_{4i} + g_{8i} + g_{9i} + g_{10,i} + g_{12,i} + g_{13,i} + g_{17,i} + g_{22,i}) \\
&(r_{ii} + r_{14,i} + r_{19,i}) (\overline{g}_{8i} + r_{14,i} + r_{19,i}) \left(\overline{g}_{12,i} + r_{14,i} + r_{19,i}\right) \left(\overline{g}_{14,i} + r_{19,i}\right) \\
&\left(\overline{g}_{19,i} + r_{14,i}\right) \left(\overline{g}_{22,i} + r_{14,i} + r_{19,i}\right) (\overline{g}_{4i} + r_{15,i}) \, \overline{g}_{15,i} \, (g_{2i} + g_{3i} + g_{7i}) \\
&(\overline{r}_{ii} + g_{7i} + g_{8i} + g_{12,i} + g_{20,i}) (\overline{r}_{ii} + g_{8i} + g_{12,i} + g_{13,i} + g_{22,i}) \\
&(r_{ii} + r_{6i} + r_{15,i}) (\overline{g}_{1i} + r_{6i} + r_{15,i}) (\overline{g}_{6i} + r_{15,i}) (\overline{g}_{8i} + r_{6i} + r_{15,i}) \\
&(\overline{g}_{9i} + r_{6i} + r_{15,i}) \left(\overline{g}_{12,i} + r_{6i} + r_{15,i}\right) \left(\overline{g}_{13,i} + r_{6i} + r_{15,i}\right) \left(\overline{g}_{15,i} + r_{6i}\right) \\
&\left(\overline{g}_{22,i} + r_{6i} + r_{15,i}\right) (\overline{g}_{2i} + r_{15,i}) (\overline{g}_{9i} + r_{15,i}) \left(\overline{g}_{13,i} + r_{15,i}\right) \overline{g}_{15,i} \left(\overline{g}_{20,i} + r_{15,i}\right) . \qquad \text{(S.17)}
\end{aligned}
$$

C.4. Horn formula

From C.2 above, one finds that

$$\Theta = \{1, 2, 3, 4, 7, 8, 9, 10, 12, 13, 17, 18, 20, 21, 22\}$$

For nodes in Θ, we use variables such as $\left(r_{3i}, \overline{r}_{3i}, G_{3i}, \overline{G}_{3i}\right)$. For node i, we still use r_{ii}. For the other nodes, we use variables such as $\left(R_{6i}, \overline{R}_{6i}, g_{6i}, \overline{g}_{6i}\right)$. We thus turn the above CNF

into the following Horn formula:

$$
\begin{aligned}
&\left(r_{ii}+\overline{R}_{6i}+\overline{R}_{11,i}+\overline{R}_{14,i}+\overline{R}_{15,i}+\overline{R}_{16,i}\right)\left(G_{3i}+\overline{R}_{6i}+\overline{R}_{11,i}+\overline{R}_{14,i}+\overline{R}_{15,i}+\overline{R}_{16,i}\right)\\
&\left(G_{4i}+\overline{R}_{6i}+\overline{R}_{11,i}+\overline{R}_{14,i}+\overline{R}_{15,i}+\overline{R}_{16,i}\right)\left(\overline{g}_{6i}+\overline{R}_{11,i}+\overline{R}_{14,i}+\overline{R}_{15,i}+\overline{R}_{16,i}\right)\\
&\left(G_{8i}+\overline{R}_{6i}+\overline{R}_{11,i}+\overline{R}_{14,i}+\overline{R}_{15,i}+\overline{R}_{16,i}\right)\left(G_{9i}+\overline{R}_{6i}+\overline{R}_{11,i}+\overline{R}_{14,i}+\overline{R}_{15,i}+\overline{R}_{16,i}\right)\\
&\left(\overline{g}_{11,i}+\overline{R}_{6i}+\overline{R}_{14,i}+\overline{R}_{15,i}+\overline{R}_{16,i}\right)\left(G_{12,i}+\overline{R}_{6i}+\overline{R}_{11,i}+\overline{R}_{14,i}+\overline{R}_{15,i}+\overline{R}_{16,i}\right)\\
&\left(G_{13,i}+\overline{R}_{6i}+\overline{R}_{11,i}+\overline{R}_{14,i}+\overline{R}_{15,i}+\overline{R}_{16,i}\right)\left(\overline{g}_{14,i}+\overline{R}_{6i}+\overline{R}_{11,i}+\overline{R}_{15,i}+\overline{R}_{16,i}\right)\\
&\left(\overline{g}_{15,i}+\overline{R}_{6i}+\overline{R}_{11,i}+\overline{R}_{14,i}+\overline{R}_{16,i}\right)\left(\overline{g}_{16,i}+\overline{R}_{6i}+\overline{R}_{11,i}+\overline{R}_{14,i}+\overline{R}_{15,i}\right)\\
&\left(G_{17,i}+\overline{R}_{6i}+\overline{R}_{11,i}+\overline{R}_{14,i}+\overline{R}_{15,i}+\overline{R}_{16,i}\right)\left(G_{20,i}+\overline{R}_{6i}+\overline{R}_{11,i}+\overline{R}_{14,i}+\overline{R}_{15,i}+\overline{R}_{16,i}\right)\\
&\left(\overline{G}_{2i}+\overline{G}_{7i}+\overline{G}_{10,i}+\overline{G}_{20,i}\right)\left(\overline{r}_{ii}+\overline{G}_{8i}+\overline{G}_{20,i}\right)\left(\overline{r}_{ii}+\overline{G}_{1i}+\overline{G}_{13,i}\right)\\
&\left(\overline{r}_{ii}+\overline{G}_{4i}+\overline{G}_{8i}+\overline{G}_{9i}+\overline{G}_{10,i}+\overline{G}_{12,i}+\overline{G}_{13,i}+\overline{G}_{17,i}+\overline{G}_{22,i}\right)\\
&\left(r_{ii}+\overline{R}_{14,i}+\overline{R}_{19,i}\right)\left(G_{8i}+\overline{R}_{14,i}+\overline{R}_{19,i}\right)\left(G_{12,i}+\overline{R}_{14,i}+\overline{R}_{19,i}\right)\left(\overline{g}_{14,i}+\overline{R}_{19,i}\right)\\
&\left(\overline{g}_{19,i}+\overline{R}_{14,i}\right)\left(G_{22,i}+\overline{R}_{14,i}+\overline{R}_{19,i}\right)\left(G_{4i}+\overline{R}_{15,i}\right)\overline{g}_{15,i}\left(\overline{G}_{2i}+\overline{G}_{3i}+\overline{G}_{7i}\right)\\
&\left(\overline{r}_{ii}+\overline{G}_{7i}+\overline{G}_{8i}+\overline{G}_{12,i}+\overline{G}_{20,i}\right)\left(\overline{r}_{ii}+\overline{G}_{8i}+\overline{G}_{12,i}+\overline{G}_{13,i}+\overline{G}_{22,i}\right)\\
&\left(r_{ii}+\overline{R}_{6i}+\overline{R}_{15,i}\right)\left(G_{1i}+\overline{R}_{6i}+\overline{R}_{15,i}\right)\left(\overline{g}_{6i}+\overline{R}_{15,i}\right)\left(G_{8i}+\overline{R}_{6i}+\overline{R}_{15,i}\right)\\
&\left(G_{9i}+\overline{R}_{6i}+\overline{R}_{15,i}\right)\left(G_{12,i}+\overline{R}_{6i}+\overline{R}_{15,i}\right)\left(G_{13,i}+\overline{R}_{6i}+\overline{R}_{15,i}\right)\left(\overline{g}_{15,i}+\overline{R}_{6i}\right)\\
&\left(G_{22,i}+\overline{R}_{6i}+\overline{R}_{15,i}\right)\left(G_{2i}+\overline{R}_{15,i}\right)\left(G_{9i}+\overline{R}_{15,i}\right)\left(G_{13,i}+\overline{R}_{15,i}\right)\overline{g}_{15,i}\left(G_{20,i}+\overline{R}_{15,i}\right).
\end{aligned}
\tag{S.18}
$$

D. The conversion of Eq. (2) into CNF form to solve feasibility problems

During the conversion one often encounters clauses like $\sum_{j=1}^{n} x_j y_j$, which can be converted into CNF form by Eq. (7) but requires exponential number of clauses (2^n). It is known, however, that there exists a transformation into CNF that avoids exponential increase in size but only preserves the satisfiability of $\sum_j x_j y_j = 1$ as the following

$$\left(\sum_j z_j\right) \prod_j \left(\overline{z}_j + x_j\right) \prod_j \left(\overline{z}_j + y_j\right) = 1, \tag{S.19}$$

where z_j (for $j = 1, 2, \cdots, n$) are auxiliary variables. Moreover, it is known that the equation $x = y$ is equivalent to

$$\left(x + \overline{y}\right)\left(\overline{x} + y\right) = 1. \tag{S.20}$$

Equations (S.19) and (S.20) allow us to convert Eq. (2) into the following CNF form

$$ABCDEFGHI = 1 \tag{S.21}$$

where

$$A = \sum_j y_j \tag{S.22}$$

$$B = \prod_{j \neq i} \left(\overline{y}_j + s_j(t)\right) \tag{S.23}$$

$$C = \prod_{j \neq i} \left(\overline{y}_j + r_{ji}\right) \tag{S.24}$$

$$D = \prod_{j \neq i} \left(\overline{y}_i + \overline{s}_j(t) + \overline{g}_{ji} + s_i(t+1)\right) \tag{S.25}$$

$$E = \overline{y}_i + \overline{s}_i(t) + r_{ii} + s_i(t+1) \tag{S.26}$$

$$F = \sum_j z_j + \overline{s}_i(t+1) \tag{S.27}$$

$$G = \prod_j \left(\overline{z}_j + s_j(t) + \overline{s}_i(t+1)\right) \tag{S.28}$$

$$H = \prod_{j \neq i} \left(\overline{z}_j + g_{ji} + \overline{s}_i(t+1)\right) \tag{S.29}$$

$$I = \overline{z}_i + \overline{r}_{ii} + \overline{s}_i(t+1) \tag{S.30}$$

and where y_j and z_j are auxiliary variables.

When the state variables $\{s_j(t)\}$ are unknown, the satisfiability of Eq. (S.21) can be in general very difficult to determine. However, if all the state variables $\{s_j(t)\}$ are known,

the satisfiability of Eq. (S.21) for the unknown edge interaction variables $\{r_{ji}\}$ and $\{g_{ji}\}$ including the auxiliary variables can be solved in polynomial time. This is because, after the substitution of the values of $\{s_j(t)\}$, only terms of the following form are left:

$$\sum_j y_j \tag{S.31}$$

$$\overline{y}_j \ (j \neq i) \tag{S.32}$$

$$\overline{y}_j + r_{ji} \ (j \neq i) \tag{S.33}$$

$$\overline{y}_i + \overline{g}_{ji} \ (j \neq i) \tag{S.34}$$

$$\overline{y}_i + r_{ii} \tag{S.35}$$

$$\sum_j z_j \tag{S.36}$$

$$\overline{z}_j \ (j \neq i) \tag{S.37}$$

$$z_i \tag{S.38}$$

$$\overline{z}_j + g_{ji} \ (j \neq i) \tag{S.39}$$

$$\overline{z}_i + \overline{r}_{ii} \tag{S.40}$$

For $j \neq i$, define new variables $Y_j = \overline{y}_j$, $Z_j = \overline{z}_j$, $R_{ji} = \overline{r}_{ji}$, and $G_{ji} = \overline{g}_{ji}$. The above terms turn into

$$\sum_{j \neq i} \overline{Y}_j + y_i \tag{S.41}$$

$$Y_j \ (j \neq i) \tag{S.42}$$

$$Y_j + \overline{R}_{ji} \ (j \neq i) \tag{S.43}$$

$$\overline{y}_i + G_{ji} \ (j \neq i) \tag{S.44}$$

$$\overline{y}_i + r_{ii} \tag{S.45}$$

$$\sum_{j \neq i} \overline{Z}_j + z_i \tag{S.46}$$

$$Z_j \ (j \neq i) \tag{S.47}$$

$$z_i \tag{S.48}$$

$$Z_j + \overline{G}_{ji} \ (j \neq i) \tag{S.49}$$

$$\overline{z}_i + \overline{r}_{ii} \tag{S.50}$$

which are in Horn-clause form because each term has at most one positive literal.

E. The proof that the Boolean process in Fig. 1(C) is feasible

We prove the feasibility of the Boolean process by actually constructing an explicit network solution (Fig. S1). We first give the necessary edge connections and ascertain that these connections are not contradictory. We then focus on each node to demonstrate that it follows the state transitions as prescribed by the process shown in Fig. 1(C).

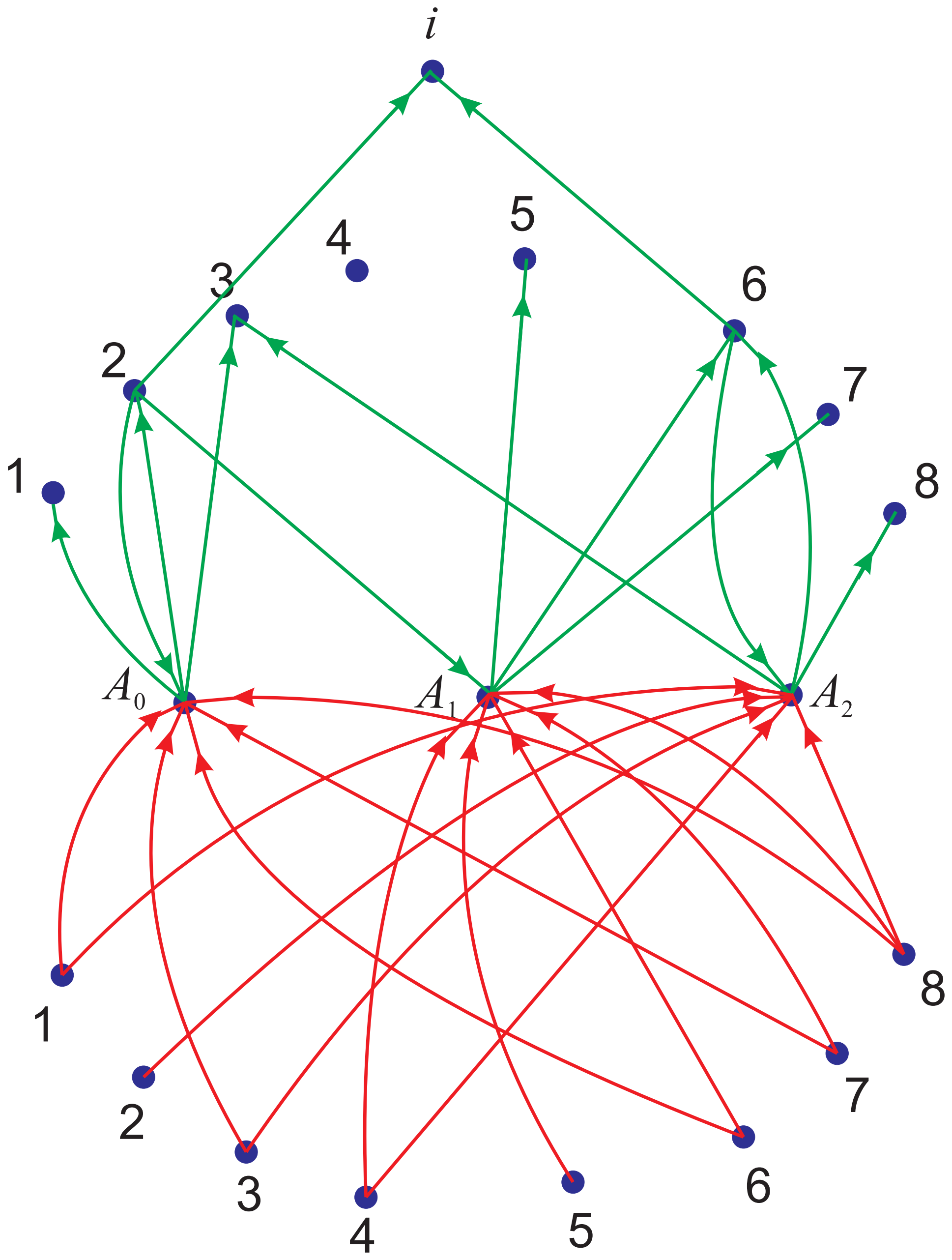

Fig. S1: An example network that can realize the Boolean process in Fig. 1(C). All the nodes are self-degradative. The red (green) arrows represent activation (inhibition). To avoid clutter, nodes $1, 2, 3, \cdots, 8$ are duplicated to separate the red arrows from the green ones.

First, we specify all necessary network connections as follows.

1. All the nodes are self-degradative. That is, every node turns off at the next time step if there is no external stimulations.

2. There are no connections among nodes A_0, A_1, A_2, and i.

3. Let θ_τ ($\tau = 0, 1, 2, 3$) denote the set of nodes $j \in \{1, 2, \cdots, 8\}$ with $s_j(2\tau) = 1$. That is, $\theta_0 = \{2, 4, 5\}$, $\theta_1 = \{1, 2, 3\}$, $\theta_2 = \{5, 6, 7\}$, and $\theta_3 = \{3, 6, 8\}$. It is required that for each τ, there is at least one node in θ_τ stimulate(s) node i.

4. For a given $\tau \in \{0, 1, 2\}$, node A_τ receives stimulation(s) from at least one node in the set θ_τ. It also receives inhibitions from **all** the nodes $j \in \{1, 2, \cdots, 8\}$ excluding θ_τ (denoted by $\overline{\theta}_\tau$).

5. For a given $\tau \in \{0, 1, 2\}$, node A_τ stimulates **all** the nodes in $\theta_{\tau+1}$.

The network in Fig. S1 satisfies the above connection requirements.

Second, we generate the dynamics according to the above network connections, starting from the initial states as given by the $t = 0$ row in Fig. 1(C). At time $t = 1$, nodes i and A_0 are turned on because of the stimulations from θ_0; the nodes in θ_0 themselves become 0 because of degradation. At time $t = 2$, the nodes in θ_1 are all turned on by node A_0; nodes i and A_0 become 0 because of degradation. Continue in this manner until the entire process is completed.

It is crucial that A_τ is turned on once and only once, for any given τ. Otherwise it would play the same role as node i and would have the same problem experienced by the process in Fig. 1(A). Our network connections guarantee that A_τ is turned on once and only once. Note that A_τ is inhibited by all the $\overline{\theta}_\tau$ nodes. In the following times defined by $t = 2\tau'$ with $\tau' > \tau$, there always exists at least one node $j \in \overline{\theta}_\tau$ that is active, which certainly prevents the re-activation of node A_τ. Otherwise, one has $\overline{\theta}_\tau \subseteq \overline{\theta}_{\tau'}$, namely $\theta_\tau \supseteq \theta_{\tau'}$. This fact implies that the Boolean equation $\sum_{j \in \theta_\tau} x_j = 1$ becomes redundant in terms of $\sum_{j \in \theta_{\tau'}} x_j = 1$. Therefore, $\sum_{j \in \theta_\tau} x_j = 1$ should have been removed in the first place.

Even though we focused on an explicit example, the construction procedure outlined in Section 4.2 and this section works for any minimal set covering problem instance.

F. Use the heuristic algorithm to find a minimal network for the Boolean process in Table S2.

The Boolean process is the same as the one in Supporting Information Section C. We still focus on node $i = 5$.

F.1. The case $r_{ii} = 1$:

In Eq. (S.16), those only containing stimulatory edges are as follows:

$$\begin{aligned} g_{2i} + g_{7i} + g_{10,i} + g_{20,i} &= 1, \\ g_{8i} + g_{20,i} &= 1, \\ g_{4i} + g_{8i} + g_{9i} + g_{10,i} + g_{12,i} + g_{13,i} + g_{17,i} + g_{22,i} &= 1, \\ g_{1i} + g_{13,i} &= 1, \\ g_{2i} + g_{3i} + g_{7i} &= 1, \\ g_{7i} + g_{8i} + g_{12,i} + g_{20,i} &= 1, \\ g_{8i} + g_{12,i} + g_{13,i} + g_{22,i} &= 1. \end{aligned}$$

By applying the greedy algorithm, we obtain minimum solutions $(g_{1i}\ g_{2i}\ g_{8i})$ and $(g_{2i}\ g_{8i}\ g_{13,i})$. Note that $(g_{1i}\ g_{2i}\ g_{8i})$ not only represents $g_{1i} = g_{2i} = g_{8i} = 1$, but also implies $g_{ji} = 0$ for $j \neq \{1, 2, 8\}$. Since $(g_{1i}\ g_{2i}\ g_{8i})$ renders the remaining equations simpler than $(g_{2i}\ g_{8i}\ g_{13,i})$ does, we choose $(g_{1i}\ g_{2i}\ g_{8i})$. After simplification, the remaining equations for inhibitory edges are:

$$\begin{aligned} r_{6i} + r_{11,i} + r_{14,i} + r_{15,i} + r_{16,i} &= 1, \\ r_{14,i} + r_{19,i} &= 1, \\ r_{6i} + r_{15,i} &= 1, \\ r_{15,i} &= 1. \end{aligned}$$

By applying the greedy algorithm again, we obtain a minimal solution $(r_{14,i}\ \ r_{15,i})$. Therefore, the minimal solution for the case $r_{ii} = 1$ is $(g_{1i}\ g_{2i}\ g_{8i}\ r_{14,i}\ r_{15,i})$.

F.2. The case $r_{ii} = 0$:

In Eq. (S.16), those only containing stimulatory edges are as follows:

$$\begin{aligned} g_{2i} + g_{7i} + g_{10,i} + g_{20,i} &= 1 \\ g_{2i} + g_{3i} + g_{7i} &= 1 \end{aligned}$$

By applying the greedy algorithm, we obtain minimum solutions (g_{2i}) and (g_{7i}). We choose (g_{7i}) since it renders a shorter form for the remaining equations:

$$\begin{aligned} r_{6i} + r_{11,i} + r_{14,i} + r_{15,i} + r_{16,i} &= 1, \\ r_{14,i} + r_{19,i} &= 1, \\ r_{6i} + r_{15,i} &= 1. \end{aligned}$$

By applying the greedy algorithm again, we obtain a minimal solution $(r_{6i} \;\; r_{14,i})$. Therefore, the minimal solution for the case $r_{ii} = 0$ is $(g_{7i} \;\; r_{6i} \;\; r_{14,i})$.

Comparing the two cases, we obtain the final minimal solution $(g_{7i} \;\; r_{6i} \;\; r_{14,i})$ for node i.

G. Budding yeast cell cycle network

Figure S2(B) is a cell-cycle network of budding yeast *S.cerevisiae* [?]. The network has $N = 11$ nodes and 34 edges. By using Eq. (2), the Boolean process is generated (Fig. S2(A)).

A

Time	Cln3	MBF	SBF	Cln1,2	Cdh1	Swi5	Cdc20/14	Clb5,6	Sic1	Clb1,2	Mcm1/SFF	Phase
t	s_1	s_2	s_3	s_4	s_5	s_6	s_7	s_8	s_9	s_{10}	s_{11}	
0	1	0	0	0	1	0	0	0	1	0	0	START
1	0	1	1	0	1	0	0	0	1	0	0	G1
2	0	1	1	1	1	0	0	0	1	0	0	G1
3	0	1	1	1	0	0	0	0	0	0	0	G1
4	0	1	1	1	0	0	0	1	0	0	0	S
5	0	1	1	1	0	0	0	1	0	1	1	G2
6	0	0	0	1	0	0	1	1	0	1	1	M
7	0	0	0	0	0	0	1	0	0	0	1	M
8	0	0	0	0	1	1	1	0	1	0	0	M
9	0	0	0	0	1	1	0	0	1	0	0	G1
10	0	0	0	0	1	0	0	0	1	0	0	G1
11	0	0	0	0	1	0	0	0	1	0	0	G1

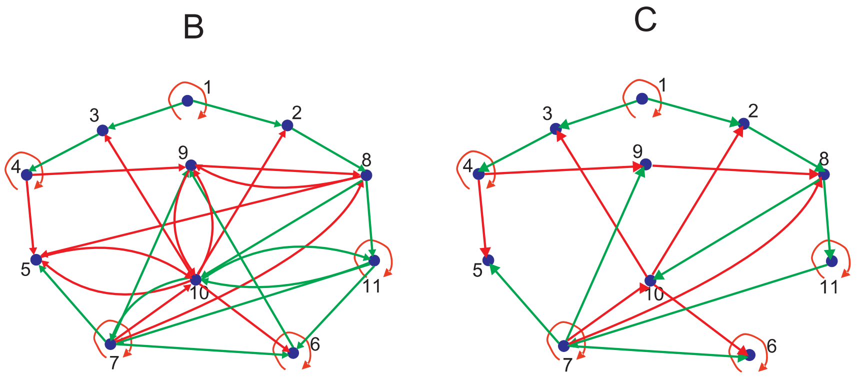

Fig. S2: Budding yeast cell cycle. (A) The time course of the 11 nodes as a representation of the cell cycle process. (B) The full network of budding yeast cell cycle, with red indicating inhibitory and green indicating stimulatory. (C) A minimal network found by the heuristic algorithm.

The process is certainly feasible, since it was generated by the network in Fig. S2(B). To

complete the other aspects of analysis, we wrote the Boolean equations and simplified them. By using Eq. (24) and the information obtained during simplification, the approximately obtained designability was 5.27×10^{27}, compared with the exact value 3.7×10^{30}.

Since the original network is known, the construction of an approximated minimal network should use edges only existed in the original network. Let r_{ji} be an edge belonging to the original network but r_{ki} not. Even though the assignment $r_{ki} = 1$ satisfies more equations than the assignment $r_{ji} = 1$, one should still select r_{ji}. As a result, this modified greedy algorithm will always yield a subnetwork of the original network, and, according to Section 3, the subnetwork has a high probability to be a genuine minimal network. If the original network contains one and only one minimal network, then the modified greedy algorithm has a high probability to obtain the genuine minimal network, although it was designed to seek an approximated one.

We applied this modified greedy algorithm many times and found that it always yields the *same* subnetwork (Fig. S2(C)). Therefore, the subnetwork may well be a genuine minimal network and it is highly likely that the original network contains no other minimal networks. Indeed, by enumerating the Boolean equations, we found that the biological process in Fig. S2(A) has in total 40,320 minimal networks, each of which has 23 edges. Among the minimal networks there is one and only one contained in the budding yeast network (Fig. S2(B)), which is exactly what was found by the modified greedy algorithm, Fig. S2(C).

---

[1] Li F, Long T, Lu Y, Ouyang Q, Tang C (2004) The yeast cell-cycle network is robustly designed. Proc Natl Acad Sci USA 101: 4781–4786.
